# A Novel Interface for the Graphical Analysis of Music Practice Behaviors

**DOI:** 10.3389/fpsyg.2018.02292

**Published:** 2018-11-26

**Authors:** Janis Sokolovskis, Dorien Herremans, Elaine Chew

**Affiliations:** ^1^School of Electronic Engineering and Computer Science, Queen Mary University of London, London, United Kingdom; ^2^Information Systems Technology and Design, Singapore University of Technology and Design, Singapore, Singapore; ^3^Institute of High Performance Computing (A*STAR), Singapore, Singapore

**Keywords:** music practice patterns, graphical user interface, visualization, music performance studies, music education, music learning

## Abstract

Practice is an essential part of music training, but critical content-based analyses of practice behaviors still lack tools for conveying informative representation of practice sessions. To bridge this gap, we present a novel visualization system, the Music Practice Browser, for representing, identifying, and analysing music practice behaviors. The Music Practice Browser provides a graphical interface for reviewing recorded practice sessions, which allows musicians, teachers, and researchers to examine aspects and features of music practice behaviors. The system takes beat and practice segment information together with a musical score in XML format as input, and produces a number of different visualizations: Practice Session Work Maps give an overview of contiguous practice segments; Practice Segment Arcs make evident transitions and repeated segments; Practice Session Precision Maps facilitate the identifying of errors; Tempo-Loudness Evolution Graphs track expressive variations over the course of a practice session. We then test the new system on practice sessions of pianists of varying levels of expertise ranging from novice to expert. The practice patterns found include Drill-Correct, Drill-Smooth, Memorization Strategy, Review and Explore, and Expressive Evolution. The analysis reveals practice patterns and behavior differences between beginners and experts, such as a higher proportion of Drill-Smooth patterns in expert practice.

## 1. Introduction

Recent advances in music computing techniques and music data capture have enable parts of music learning to be supported by personal computers and smart mobile devices. Software applications such as Wolfie,^1^ Superscore,^2^ and BandPad^3^ score follow students whilst they play and mark notes played as correct or incorrect. Some music education software also track time spent on practizing a piece. These applications focus on improving students' technical abilities, but lack the capability of monitoring the development of expressivity, a defining aspect of musical performance. To fill this gap, we introduce the Music Practice Browser (MPB), which allows users to monitor the development of expressivity in music practice.

Music tutoring applications such as iScore (Upitis et al., 2012) and PRAISE^4^ offer human tutoring support and assessment through online graphical user interfaces; tutors can annotate user-provided recordings and send feedback to students to guide further practice. Other interfaces have introduced visual feedback to help music learners correct errors in pitch accuracy, for example, in singing (Huang and Chu, 2016). Some use gamification and traveling metaphors to engage users to think about music performance. In the Jump'n'Rhythm (Alexandrovsky et al., 2016) video game, the user has to make a virtual character jump in time to rhythm patterns. The Expression Synthesis Project (ESP) interface uses an automobile driving (wheel and pedals) to modulate tempo and loudness (Chew et al., 2005), with roadmaps acting as guides to expressive performance (Chew et al., 2006). Such interfaces extend traditional teaching techniques, but do not provide an overview of whole practice sessions. MPB offers novel Practice Session Work Maps of entire practice sessions and Practice Segment Arcs to allow music teachers and learners to visually assess practice strategies to overcome specific difficulties.

In order to test the efficacy of the proposed visualizations, the MPB framework is tested on recorded practice sessions of beginner, intermediate, and expert piano learning students. We show that the MPB framework allows us to clearly observe important practice behaviors, including Drill-Smooth and Drill-Correct, a Memorization Strategy, Review and Explore behavior, and Expressive Evolution. These practice patterns are coined by the authors and are explained in detail in Section 5. The patterns are statistically analyzed in this empirical study.

The article is organized as follows: Section 2 gives a review of related visualization techniques in the literature; Section 3 describes how these visualization techniques are altered to fit within the MPB framework; Section 4 describes an empirical study; Section 5 describes practice patterns observed from the study; and, finally, Section 6 concludes the article with a discussion of the results and future directions.

## 2. Existing music practice visualizations

This section gives an overview of existing visualization techniques for representing and evaluating music practice behaviors, with focus on techniques most directly related to the MPB.

### 2.1. Practice session maps

**Figure 1:**
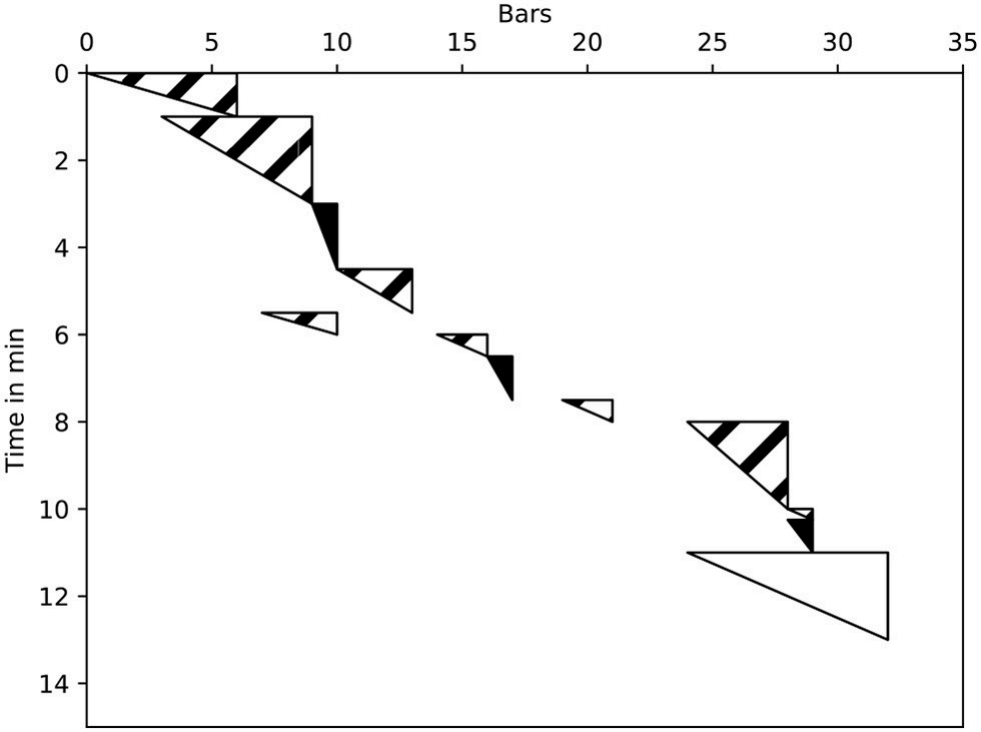
Distribution of musical material and subject's activity in time; illustration based on Miklaszewski (1989).

Practice Session Maps (PSMs) were first proposed by Miklaszewski (1989). The graphical form they used showed practice activity as a distribution of music material over time. The horizontal axis represented the score measure and the vertical axis practice time. A practice segment is defined as any continuous playing of part of the score, which ends wherever the player stops, even briefly. Color-coded triangles indicate the speed at which the segment was played: black indicates slow playing; white marks segments played at the final tempo; and, stripes depict irregular tempo changes. This is depicted in Figure 1.

Other PSMs are proposed in Chaffin and Logan (2006), Chaffin et al. (2003), and Chaffin et al. (2010), in the context of studying memorization strategies and the acquisition of expert knowledge. By observing practice sessions, practice segments were categorized as “explore,” which included shorter practice segments, and ‘smooth out/listen', comprising of longer practice segments.

The MPB's Practice Session Work Map (PSWM) builds on and expands on these PSM representations. The MPB additionally provides visualization of practice segments in relation to the formal structure of the piece. Practice patterns are also called out, and color-coded in the graphs.

### 2.2. Arc diagrams

MPB uses Practice Segment Arcs to identify problematic segments—segments that require more work—in a piece, allowing users to view the practice segments described in the previous section on one continuous axis. Although arc maps have been used in a number of extra-musical contexts, and even in the the visualization of music structure, this represents the first use of arc maps in the context of music practicing.

**Figure 2:**
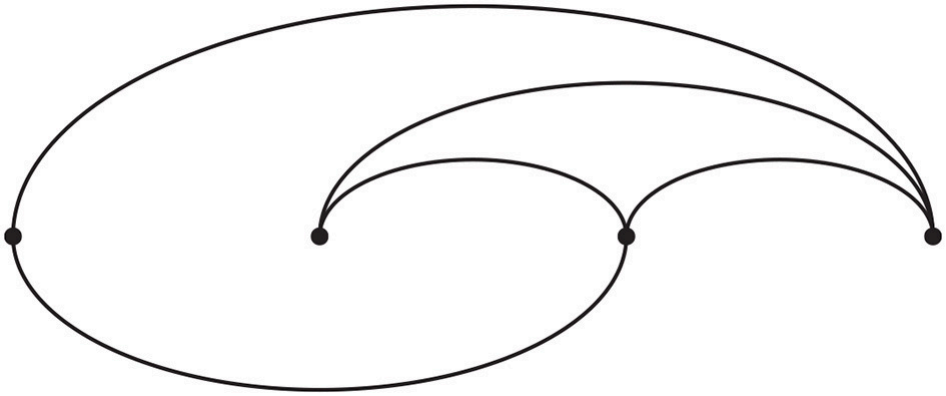
Arc diagram; illustration based on Nicholson (1968).

One of the first uses of arc diagrams was presented by Saaty (1964) and Nicholson (1968) in the field of combinatorics. In these studies, the authors connected nodes positioned on a single axis by using arcs with the least amount of crossings. This is depicted in Figure 2. Kerr (2003) used arc diagrams to represent email threads, which allowed for easy viewing of the chronology of messages. Derivatives of this visualization technique are used in text analysis; for instance, Don et al. (2007) and Collins et al. (2009) showed that arc diagrams can be useful in text mining, by visualizing repeated words and their frequency.

Wattenberg (2002) used arcs to represent complex patterns of repetition in sequential data. In the context of music, a form of sequential data, arc diagrams have been used to illustrate different music structures. Schankler et al. (2011) and Smith et al. (2014) used arc diagrams to represent formal music structures. In OMax (Assayag and Dubnov, 2004) and Mimi (Francois et al., 2011) human-machine improvisation systems, arc maps show connections between similar (and continuable) transitions between segments of music in the factor oracle. In Lamere (2014), parts of a song that sound good together are connected via an arc diagram in the “*Autocanonizer,”* which makes a canon out of commercially available music.

MPB presents a novel use of arc diagrams, using them to represent repeated segments in a practice session. In so doing, parts of the music that a player finds difficult or challenging will show up as densely repeated sections.

### 2.3. Tempo-loudness diagram

Expressivity can be quantified through variations in tempo and loudness, amongst other parameters. Tempo-loudness graphs provide an easy way of analysing and comparing expressive music performances. See, for example, (Langner and Goebl, 2003; Widmer et al., 2003; Kosta et al., 2014).

Tempo and loudness information are often extracted from audio data. Tempo information is obtained by detecting (or manually annotating) each beat, then taking the inverse of the time difference between these beats. Musical beats can be automatically detected using audio beat detection software (Laroche, 2003; Ellis, 2007). When the score exists, we can use dynamic time warping to align a recording to the score to determine beat positions (Hu et al., 2003). However, significant error can accrue when using such automatic methods. The interested reader is referred to Gouyon et al. (2006) for a review of automatic tempo extraction techniques. Loudness is often quantified in sones, a unit of perceived loudness first introduced by Stevens (1936). It is important to consider perceived loudness because, for example, low frequencies must be sounded at higher dB levels to achieve the same perceived loudness as higher frequencies at lower dB levels. Measurements in sones account for that difference. Furthermore, sones provide a linear scale for measuring loudness, i.e., doubling the sone value produces a sound that is perceived to be twice as loud.

The IMMA system developed by Herremans and Chuan (2017) visualizes tension characteristics (as defined by Herremans and Chew, 2016) of both audio and score in sync with the score and audio performance. While the system is not specifically designed for an educational setting, the tension characteristics include loudness. Langner and Goebl (2003) introduced a method for displaying both tempo and loudness variations derived from expressive music performances in a two-dimensional space: tempo on the x-axis and loudness on the y-axis. Using this representation, Widmer et al. (2003) clustered and visualized different types of expressive gestures, which were used to differentiate performers one from another. Dixon et al. (2002) used this two-dimensional representation in their interactive conducting interface, the ‘performance worm'. Examining tempo and loudness shapes in relation to music structure (phrases, sections), Windsor et al. (2006) and De Poli et al. (1998) suggest methods for separating, profiling, and quantifying the contributions of different structural components to tempo and loudness. In MPB, Tempo-loudness graphs will allow the user to visualize and monitor the development of expressivity through the practice sessions.

### 2.4. Piano roll notation showing right/wrong notes

The piano roll originally consisted of hole-punched paper used for triggering actions in mechanical player pianos (Hosley, 1910; Dolge, 1911). It persists today in music games like guitar hero, where the player must strike notes that roll over the screen at the appropriate times. It is widely used in sequencing software such as LogicPro,^5^ Ableton Live,^6^ and Linux MultiMedia Studio (LMMS)^7^ to visualize and edit music.

Few representations of piano-roll representations exist in the context of musical learning. Synthesia^8^ uses a scrolling piano roll to display notes to be played; the player has to hit the notes as they approach the play bar. The interface described by Fernandez et al. (2016) additionally uses an Augmented Reality system to show an interactive character that gives real-time piano-roll-based feedback during playing. Yousician (Kaipainen and Thur, 2015) uses a scrolling guitar roll to tell the player when to strike each note. After the performance is finished, the system displays which notes have been played correctly and which have not.

In the realm of music transcription, Benetos et al. (2012) demonstrated the performance of their score-informed transcription algorithm using a piano roll representation: black notes signal correctly-played notes, gray marks missed notes, and empty rectangles indicate extra notes struck by the player. Piano roll representations have also been used to model music in a more abstract way. Research on using deep neural networks for modeling transition probabilities in music have been shown to work reasonably well when using piano-roll representations (Boulanger-Lewandowski et al., 2012; Sigtia et al., 2016). Tsubasa et al. (2015) proposes a visual representation of a performance using piano-roll with correctness markings as an indicator of the technical ability of the piano player. They further suggest a method for simplifying the score based on the player's ability to play correct notes. The bagpipe chanter learning interface described by Menzies and McPherson (2013), uses a piano-roll to compare the performances between a tutor and a learner.

MPB implements a piano-roll representation augmented with markers for correct and incorrect notes (i.e., Practice Session Precision Map) to visualize an entire practice session. In the next section, we expand on the four existing visualization techniques discussed in this section to describe how they are implemented in MPB.

## 3. New visualizations of piano practice

This section describes how techniques from existing literature are augmented and integrated into a usable tool for analysing musical practice behavior. The section is organized as follows: first, we describe how we augment practice session maps to afford greater visual detail on the practice session, thus forming Practice Session Work Maps; we introduce Practice Session Precision Maps with indicators of note correctness over a whole practice session; we then describe how arc representations are expanded to visualize the structure of a practice session and highlight challenging sections in the score using Practice Segment Arcs; lastly, we describe how traditional tempo/loudness graphs can be leveraged to monitor the evolution of expressivity throughout a practice session through Tempo-Loudness Evolution Graphs.

### 3.1. Practice session work maps

**Figure 3:**
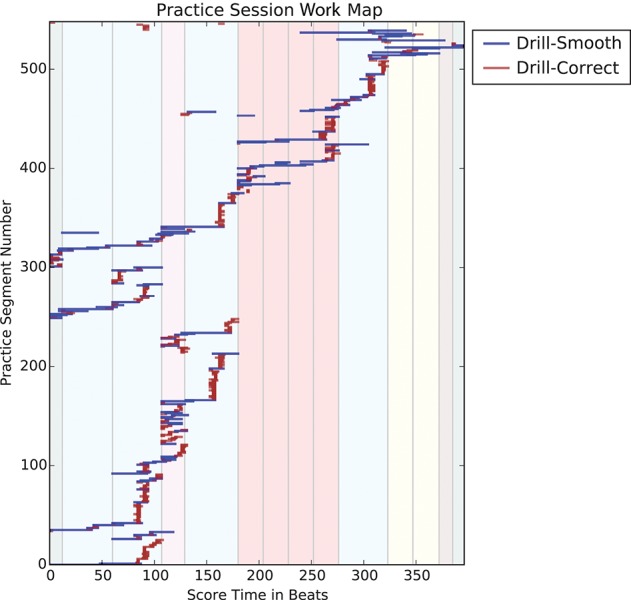
Practice Session Work Map of an expert player (E-1)'s hour-long practice of Chopin's “Mazurka in A minor, Op. 17 No. 4,” with Drill-Correct and Drill-Smooth sections marked in dark red and blue, respectively. Color panels in the background show the formal structure of the piece.

Practice Session Work Maps (PSWMs) are an extension of the PSMs. PSWMs augment PSM by highlighting in color two different types of practice behaviors: Drill-Smooth and Drill-Correct. Figure 3 shows a Practice Session Work Map derived from a practice session in the experiment described in the next section. A practice segment is defined as any continuous playing of part of the score, which ends wherever the player briefly stops. Each horizontal line in Figure 3 represents a practice segment. The x-axis represents the length of the music piece, expressed in beats. The y-axis represents a count of the practice segments in a practice session. Colored vertical panels in the background mark sections in the piece; similar sections are coded using the same colors, showing the formal structure of the piece.

A key extension of PSM is the encoding of two predominant types of drill practice patterns: *Drill-Correct* and *Drill-Smooth*. Drill-Correct (coded dark red) is a kind of practice pattern typically only a few beats long; here, the player is working on specific technical skills such as interval leaps, fingerings, and note correction. This practice pattern shows up as a vertical stack of short dark red lines. Drill-Smooth forms the second type of practice pattern. These and other patterns are discussed in Section 5.

### 3.2. Practice session precision map

As described earlier, piano-rolls constitute a useful tool for visualizing the player's technical proficiency. We first describe the piano-roll representation augmented with accuracy information (part of MPB) before introducing the Practice Session Precision Map (PSPM).

**Figure 4:**
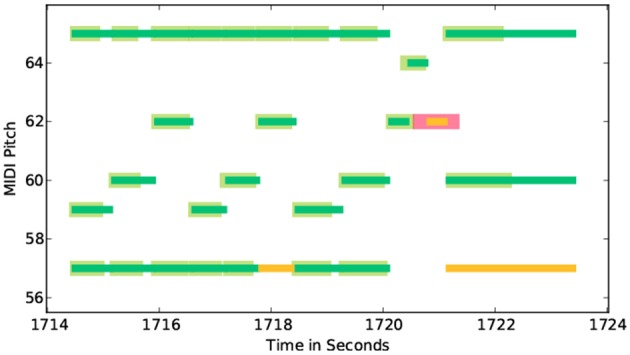
Piano-roll representation augmented with playing accuracy information for segment No.313 from expert player (E-1). Thin green lines represent notes from the score, thick green lines notes played, thin yellow lines skipped notes, thick red lines incorrect notes.

Figure 4 shows the augmented piano-roll representation displaying note correctness for a practice segment. The thinner horizontal lines represent pitches extracted from the score. Correctly played notes are marked green; missed notes are marked yellow. Thicker green lines represent notes played by the student; correct ones are colored green and incorrect ones red. In this particular example, the yellow lines at the bottom indicate that the player missed these (low) notes. The thicker red line marks a right note played at the wrong time.

**Figure 5:**
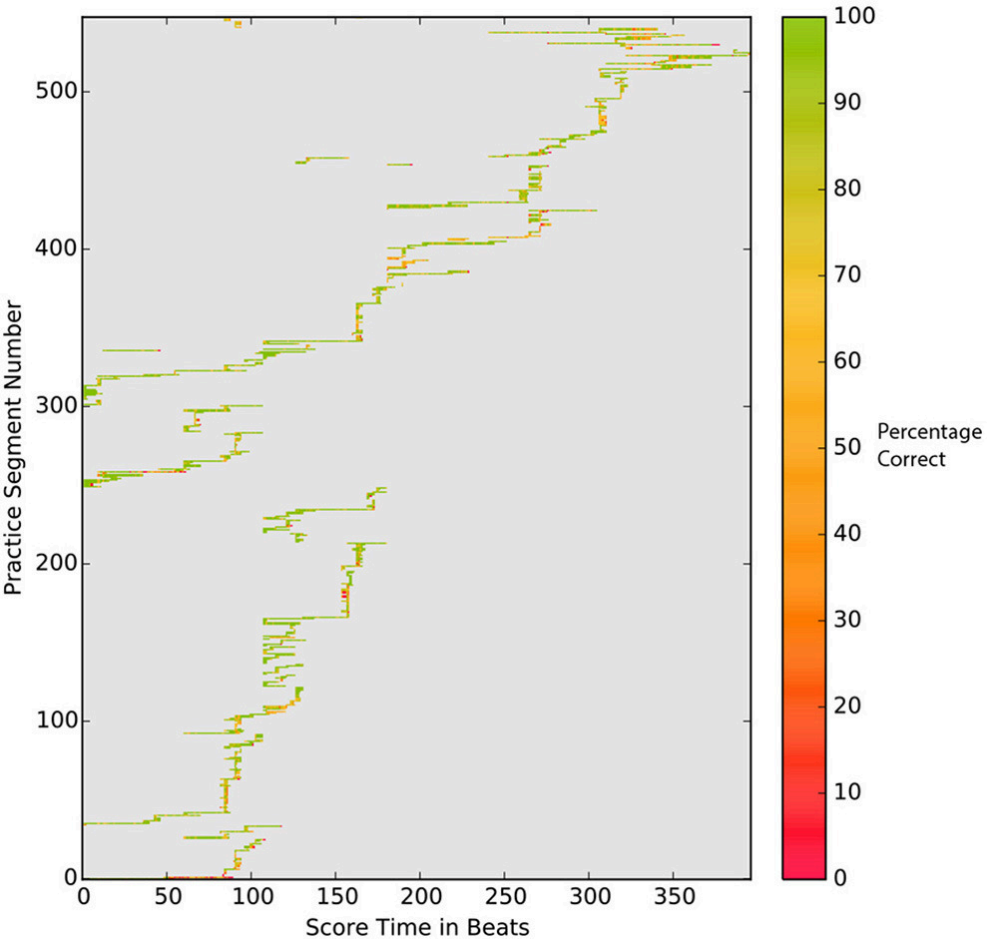
Practice Session Precision Map for expert player (E-1). Graph shows coverage for each beat; the color marks note accuracy beat (% correct) within each beat.

While the above piano-roll representation provides an informative view of playing accuracy, it is not easy to relate this to the practice session as a whole. In the PSPM, we encode the average note accuracy per beat using color as shown in Figure 5. This representation gives a clear and detailed overview of the notational accuracy, particularly how it varies over the practice period.

### 3.3. Practice segment arcs

**Figure 6:**
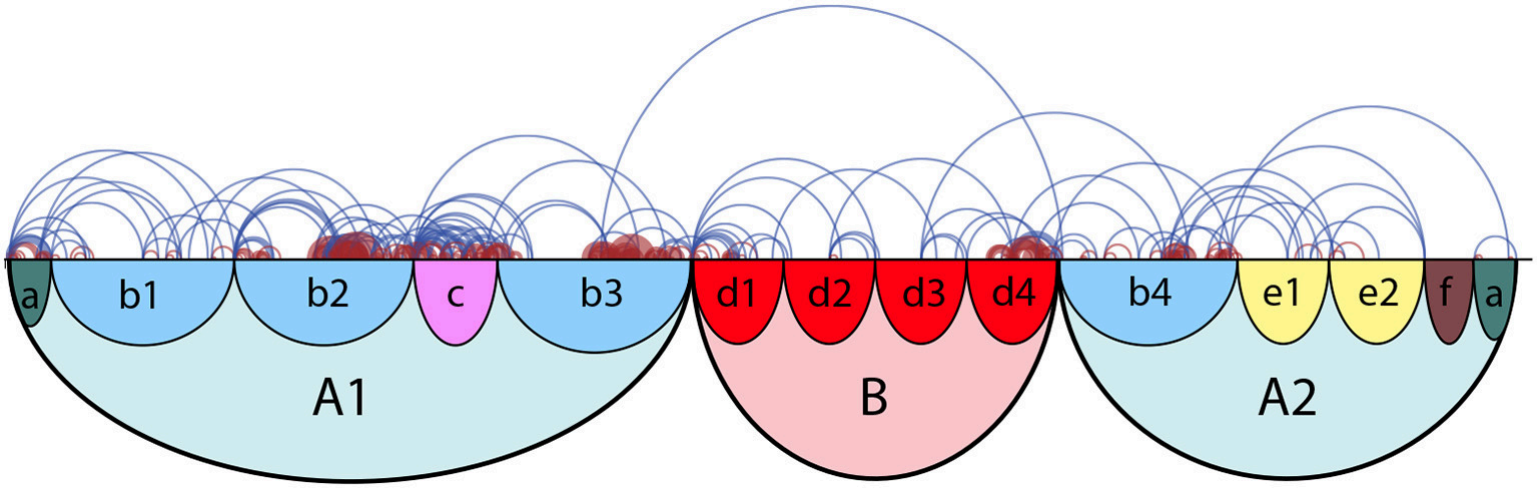
Illustration of Practice Segment Arcs: Practice Segment Arcs for expert player E1 (upper half) with formal structure of piece (lower half).

Arc diagrams are often used to represent structural connections between entries in a large dataset. In the MPB framework, arc diagrams show the links between different practice segments. The top half of Figure 6 shows a Practice Segment Arc (PSA) visualization based on the practice session of an expert player in the empirical study. The start (left side) of an arc represents the start of a practice segment, and end (right side) of the arc marks the end of a practice segment. Like in Practice Session Work Maps, the arcs are color-coded to represent Drill-Correct segments (dark red) and Drill-Smooth segments (blue).

PSAs can be used to visually identify difficult sections within a piece or to observe the connections between practice segments, all on one single axis. The horizontal axis represents the length of the piece. When PSAs are juxtaposed on the formal musical structure, such as that in Schankler et al. (2011), they can be used to reveal difficult passages in relation to the formal structure of the piece (see Figure 6). The formal structure of this example is explained in Section 4.1.3.

From this figure we can observe that a lot of practice time was spent linking subsections *b2* and *c*. This example also demonstrates that the student focused a lot of effort on the second part of subsection *b3*, and on linking subsection *d4* with *b4*. In the next section, we focus on how we can further investigate these places of interest with the use of Tempo-Loudness Evolution Graphs.

### 3.4. Tempo-loudness evolution graphs

Tempo-Loudness Evolution Graphs (TLEGs) allow us to observe how expressivity evolves over the course of a practice session. This is achieved by color coding tempo graphs and loudness graphs. Independent interviews with four experts revealed that participants found it easier to understand graphs where only tempo or only loudness are presented. As discussed in Section 2.3, Langner and Goebl (2003) proposed the performance worm visualization that showed tempo and loudness on the same graph. Although this is a useful visualization tool, we separated tempo and loudness in the graphs to make them more understandable to our participants, who have not had much training on how to read graphs.

**Figure 7:**
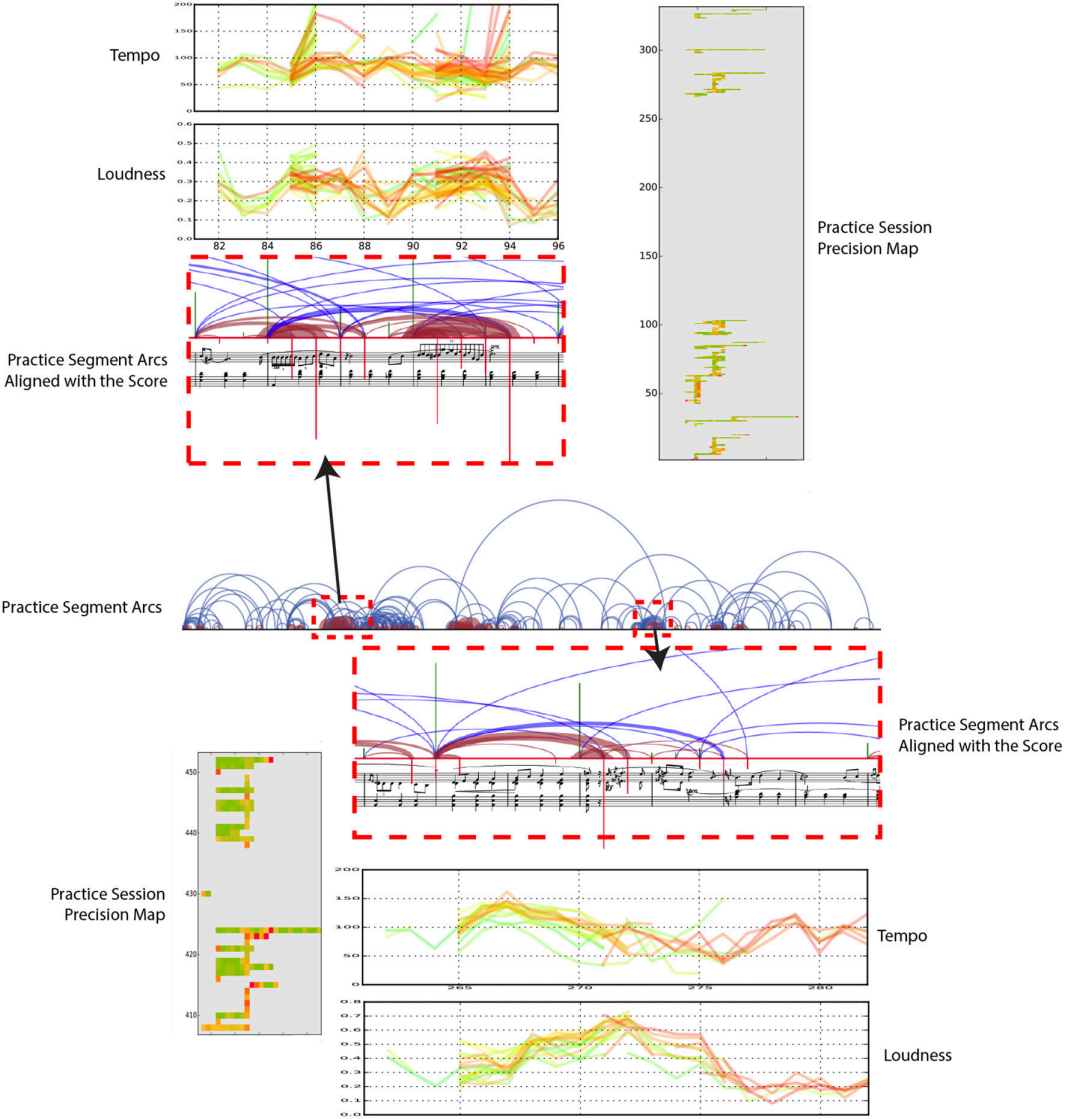
Tempo-Loudness Evolution Graphs (top left and bottom right) for expert player (E-1). Green lines depict tempo/loudness at beginning, progressively turning to red by end, of practice session. The x-axis marks the time within the piece (score time). Also shown are Practice Segment Arcs and Practice Session Precision Maps.

TLEGs can be seen in the top left and bottom right in Figure 7. The figure also includes a Practice Session Precision Maps and Practice Segment Arcs as a demonstration of how these graphs can be used in a mutually-informing fashion.

TLEGs use a gradual color coding that changes from green (start of the practice) to red (end of the practice) to track performed loudness (normalized MIDI) and tempo (beats per minute) throughout the practice in score time. Figure 7 zooms in on two dense regions in the Practice Segment Arcs (red dashed rectangles) to examine practice segments that have been repeated numerous times (measures (mm.) 28–32 and mm. 88–93). The corresponding TLEGs reveal how the expressiveness changes throughout the practice, as measured by tempo and loudness variations.

**Figure 8:**
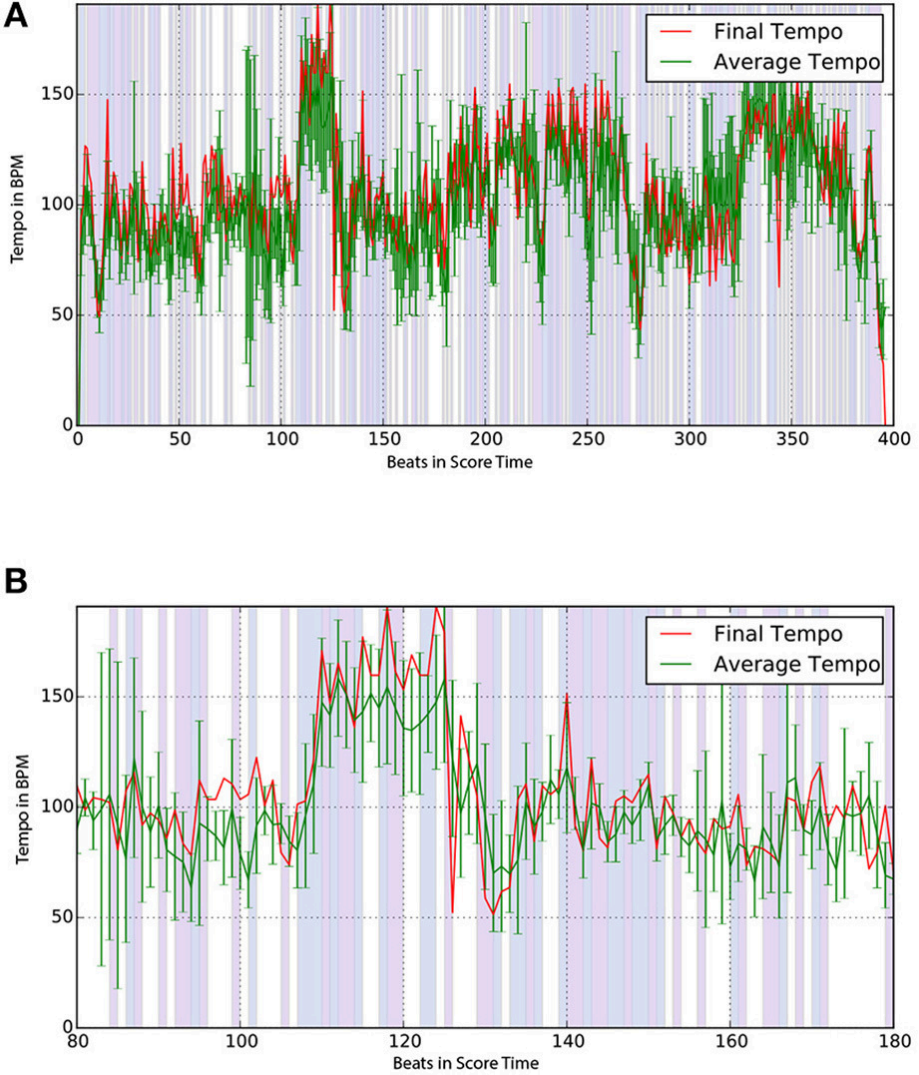
Final (red) and average practice tempo (green) for an expert player (E-1). Backgrounds show same (decrease=light red, increase=light blue) and different (white) tempo changes. **(A)** Average and final tempo of full performance for an expert player (E-1). **(B)** Zoomed in version of (a) for time 80s to 180s.

On the occasions when the participant was able to play the musical piece from beginning to the end we asked the participant to perform the piece at the end of the practice session. Figure 8 shows the comparison between the average tempo derived from practice sessions and the final tempo of the final performance (E-1). The graphs show that the two curves have a very similar profile, with the final tempo slightly faster than the practice average. Non-white areas in the background mark tempo changes in the same direction between the practice average and the final tempo plots; white areas indicate different directional changes. Light-blue areas indicate an increase in tempo in both graphs, light-red areas indicate a decrease.

In this section, we have described a number of new visualization techniques implemented in MPB for use in a piano learning environment. These graphical tools enable users to extract and gain insights into different practice behaviors. The next section outlines an experiment designed to identify practice behaviors using these visualization tools.

## 4. Empirical study

In this section, we describe an empirical experiment in which the MPB is used to identify common practice behaviors of piano students of varying competency levels: expert, intermediate, and beginner. The study will demonstrate how MPB provides different views of recorded musical practice sessions and allows users to visually inspect and analyse music practice sessions.

### 4.1. Experimental setup

Here, we describe how the experiment was set up. First, we describe the participants, then we describe how the data was collected, and finally the music that was used in the experiment.

#### 4.1.1. Participants

A total of eight participants were asked to practice a piano piece new to them for one hour. The group of participants included two concert pianists (referred to as experts E-1 and E-2), four intermediate pianists who occasionally play the piano for fun (I-1 through I-4), and two novices who are beginner pianists and have rudimentary sightreading skills (N-1 and N-2).

#### 4.1.2. Procedure

The practice sessions, conducted on a Yamaha HQ 300 SX Disklavier II, are recorded using a set of stereo microphones installed above the piano. The MIDI output was recorded on a laptop and loudness information obtained from MIDI note velocities. Practice segment starts and stops were manually annotated, by the first author, using Sonic Visualizer (Cannam et al., 2010). Annotation accuracy was checked aurally and visually by superimposing the annotations, MIDI, and audio. Note asynchrony in played chords pose a challenge in annotating live piano performances. In instances where chords were played asynchronously, the median of the note onsets was taken to be the chord onset time. The annotations were exported in CSV format, for easy parsing by MPB. The visualizations and analyses implemented in MPB are coded in Python with the use of the *Matplotlib* library (Hunter, 2007). The score of the piece is parsed using the *music21* toolkit (Cuthbert and Ariza, 2010).

#### 4.1.3. Practice piece

The piece selected for the experiment was Chopin's “Mazurka in A minor, Op. 17, No. 4”, which is used in its entirety, 132 measures with 396 beats. In the experiment to be described, practice sessions using this piece are conducted over an hour each to provide a rich and ecologically valid dataset of significant size for analysis and investigation. Rather than aiming for general results that would be difficult to apply to any individual piece, we have chosen to give an in-depth case study of one classical piece, with extended recorded practice sessions.

**Figure 9:**
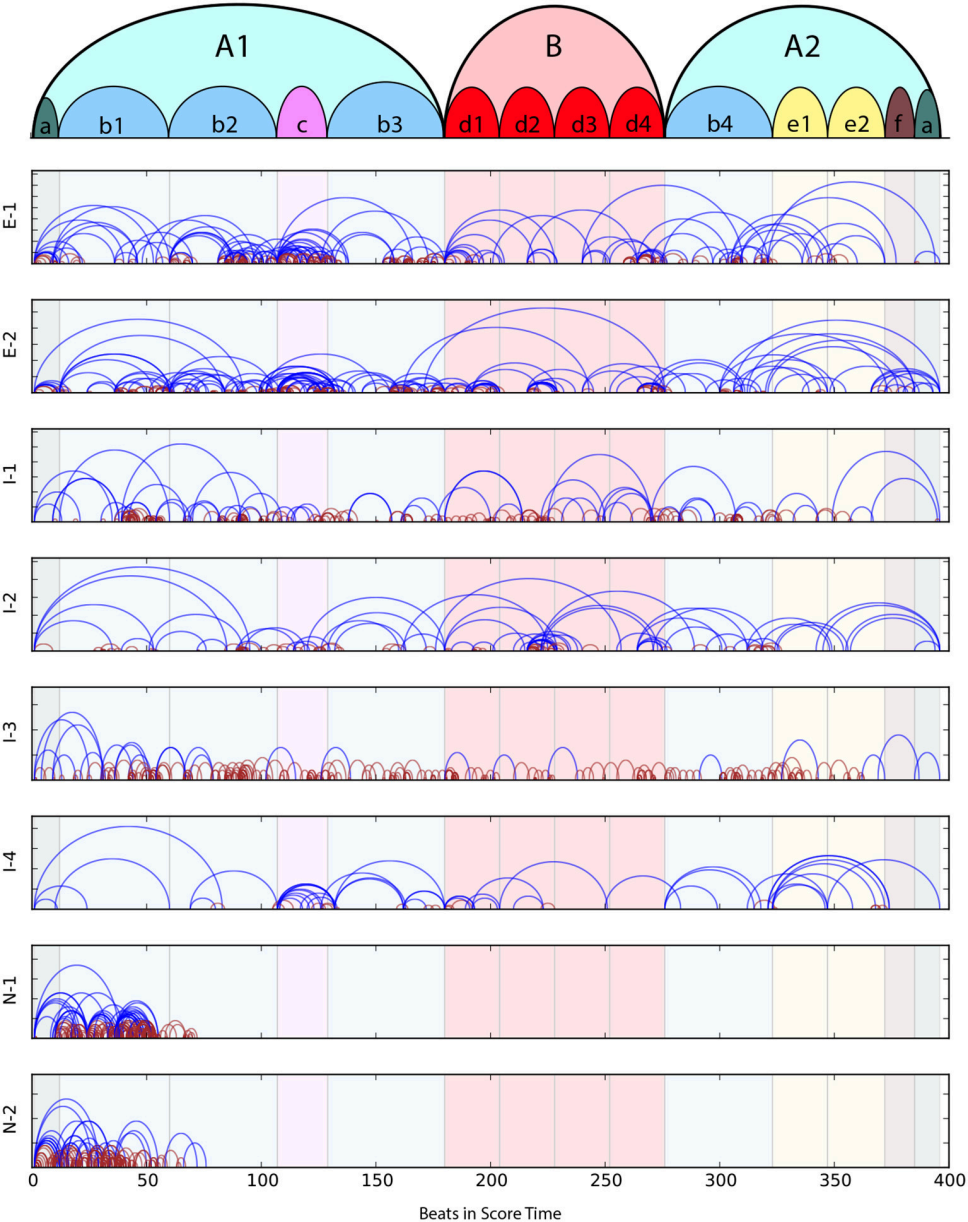
Practice Segment Arcs for each participant. The top-most diagram shows the sectional structure of Chopin's “Mazurka in A minor Op. 17 No. 4.” In subsequent graphs, blue lines mark Drill-Smooth segments and dark red lines are Drill-Correct segments; background panels delineate the formal structure of the piece.

The piece itself can be divided into three main sections and 14 subsections. The top-most arc diagram in Figure 9 shows the sectional structure of the piece at two hierarchical levels. This figure closely follows Pittman's (Pittman, 1999) Schenkerian analysis (Schenker, 1925) of the work. The piece is notated in 3/4 time and tends to last between 3 to 6 minutes in performance, depending on the tempo chosen by the performer. The marking at the beginning of the piece '*Lento ma non troppo'* indicates that the piece should be slow, but not too much. For the 46 recordings of this mazurka in the MazurkaBL dataset Kosta et al. (2018), the average tempo is 104.83 BPM, with standard deviation of 39.3 BPM.

This piece is chosen because it has a variety of different challenges for pianists of different levels. The piece is structured in sections with repetitions that allow repeated parts to be compared. The most difficult parts of the piece require a high level of proficiency, which allows the most advanced players to display how they tackle such passages. These challenging sections also provide interesting test material to compare expert behavior with novice practice.

### 4.2. Overall practice behavior

Figure 9 shows the Practice Segment Arcs for each participant; blue for Drill-Smooth (DS) segments and dark red for Drill-Correct (DC) segments. Colored panels in the background display the formal structure of the piece is given at the top of Figure 9. The descriptions below give a brief overview of the practice behaviors observed using MPB's Practice Segment Arcs (PSAs):

E-1 Many repetitions of short DS and DC segments in subsections *b2* and *c*; above average DS (dark red arcs) coverage at transitions into and out of section *B*; remainder of session focussed on DS behaviors and connections between subsections.
E-2 Dense coverage of primarily DS arcs at transitions between *b1* and *b2*, around *c*, within *b3* and *d1*. Practice segments tend to be longer than E-1's, in particular, entire section *B* is covered in one long DS arc.
I-1 A few segments without DS activity (blue arcs); dense DS behavior (dark red arcs) in small parts of most subsections except for the beginning and end of the piece, but these are sometimes not followed by DS behaviors.
I-2 Mainly long Drill-Smooth arcs with few DS segments; considerable time spent on transition between subsections *d2* and *d3*.
I-3 Mainly DS segments (short dark red arcs); DS segments, which typically follow drill behaviors, are infrequent.
I-4 Mainly long DS segments, with denser coverage in subsections *c, e1*, and *e2*.
N-1 Focused on the first 2+ subsections; many short DS segments, with a good number of DS segments, but only at beginning of piece.
N-2 Similar to N-1, covering only the first 2+ subsections, but with shorter DS arcs.

**Figure 10:**
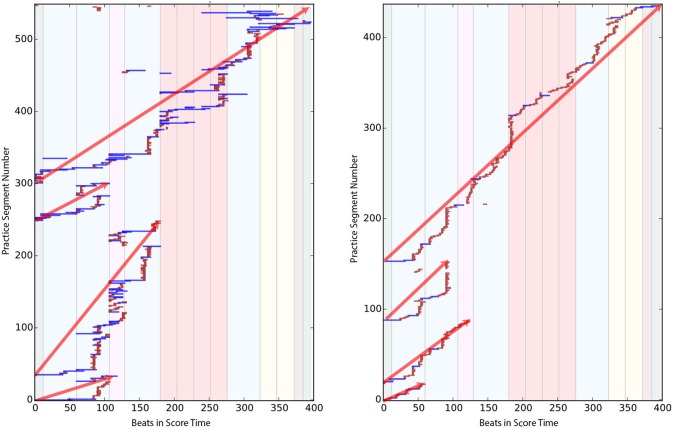
Practice Session Work Maps of E-1 **(Left)** and I-3 **(Right)** that reflects the tendency to play from start to end of the piece (red diagonal line).

In all of the practice sessions, players worked progressively from the beginning of the piece to the end. Figure 10 shows the Practice Session Work Maps for E-1 and I-3; this tendency is highlighted with diagonal red lines.

Visual inspection of the Practice Session Work Maps show that intermediate players often practice large portions of the piece without stopping, more so than experts, who more frequently pause to work on specific areas. This is reflected in Figure 10, where, the expert practice behavior (left) shows more pauses, repetitions and even non-sequential playing (i.e., jumping to other sections) in the practice session. We presented our collected data to four independent experts, who were able to identify two experts out of two correctly, based solely on our graphical representations.

In the next sections we will further examine and quantify this behavior and identify a number of observed practice behaviors through MPB's visualizations.

## 5. Observed practice patterns

We use MPB's visualizations to analyse practice behaviors and patterns. This section describes the observed common practice patterns, including drill strategies (DC and DS), Review and Explore behaviors, and the Evolution of Expressivity.

### 5.1. Drill strategies

The new MPB visualizations allow us to identify two new common practice patterns in the practice sessions: Drill-Correct (DC) and Drill-Smooth (DS). For clarity, we will refer to these patterns as drill clusters.

#### 5.1.1. Drill-correct

**Figure 11:**
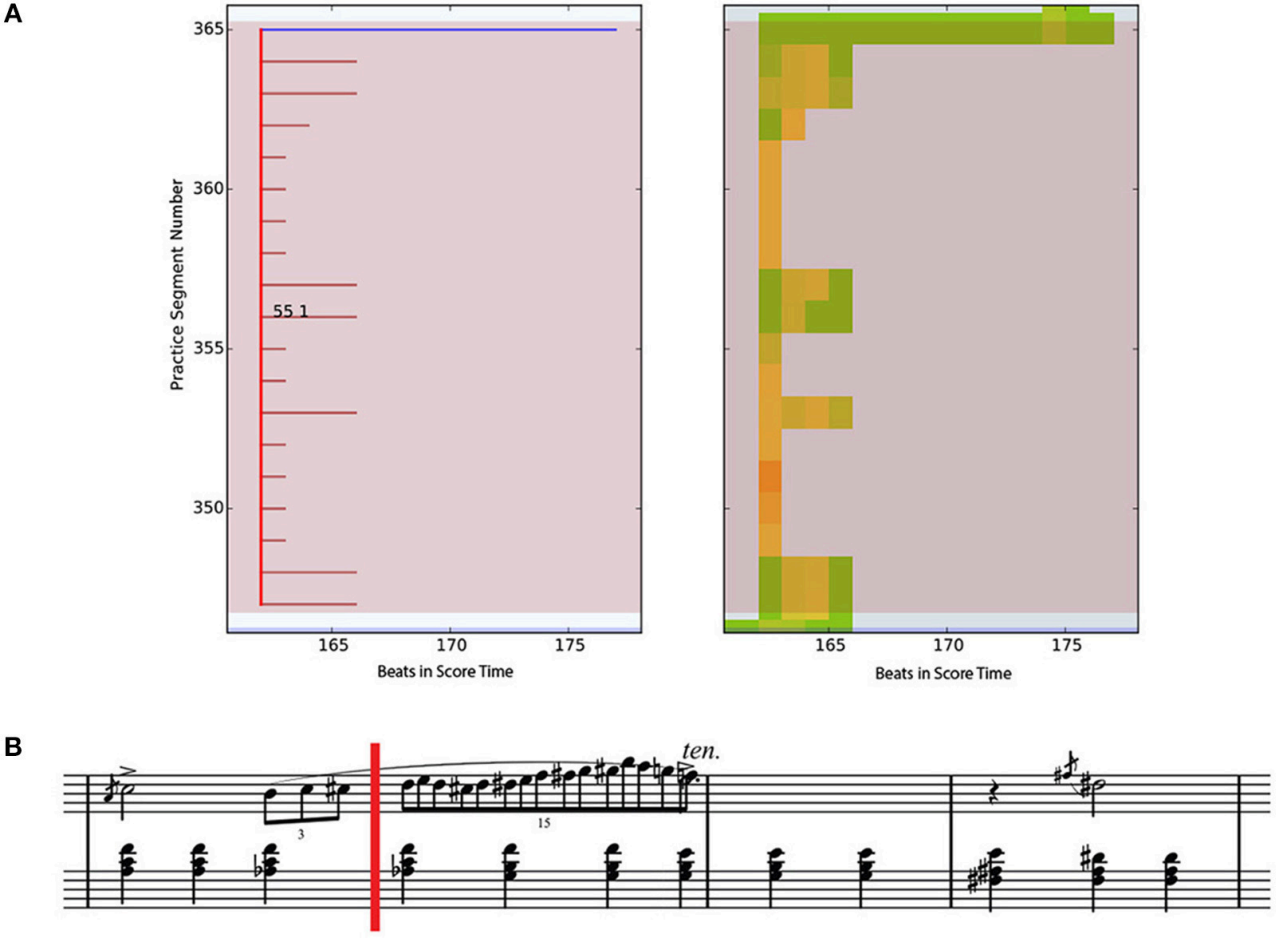
Practice Segment Work Map (top left) and Practice Segment Precision Map (top right) for a Drill-Correct practice pattern by E-1; corresponding music segment is shown in the score (lower part of figure). **(A)** Left - Practice Session Work Map with color annotations of drills (dark red) and smooth (blue). Right - Practice Session Preci-sion Map with color annotations of correct (green) and incorrect (red) beats. **(B)** Corresponding score. The point of interest starts at the red vertical line (m. 55 beat 1).

Figure 11 shows a typical DS pattern. On the left-hand side of the figure, a Practice Session Work Map (PSWM) is displayed, which shows the DS (dark red) and DS (blue) segments. In this drill cluster, all segments start from the same beat. The participant is working on an isolated part of the piece, as displayed in the second bar (fast paced passage) of the corresponding score in Figure 11B.

The right-hand side of the figure shows the Practice Session Precision Map (PSPM). As described previously PSPMs indicate note correctness within the practice session. Each small square on the map corresponds to one beat in the score. We can observe many yellow beats in the PSPM, indicating that about half of the notes in the beat were played correctly. It is also worth noting that the participant performed better in the middle of the cluster than at the beginning (bottom of the graph). At the last practice segment in the cluster (top line), the participant plays through the section with hardly any mistakes.

The PSWM calculates DS clusters by going through the practice session in a sequential manner until the first drill segment (i.e., a segment which has less than 10 beats) is found at time *t*_*i*_. For each consecutive segment we check if there is a drill segment that starts at time *t*_*j*_ where *i*−3 < *j* ≤ *i*. As long as the 'attached' segments comply with these rules, they are added to the cluster. When a longer segment is detected that starts at time *t*_*j*_, with *i*−3 < *j* ≤ *i*, the cluster ends and we have identified a DS cluster.

#### 5.1.2. Drill-smooth

**Figure 12:**
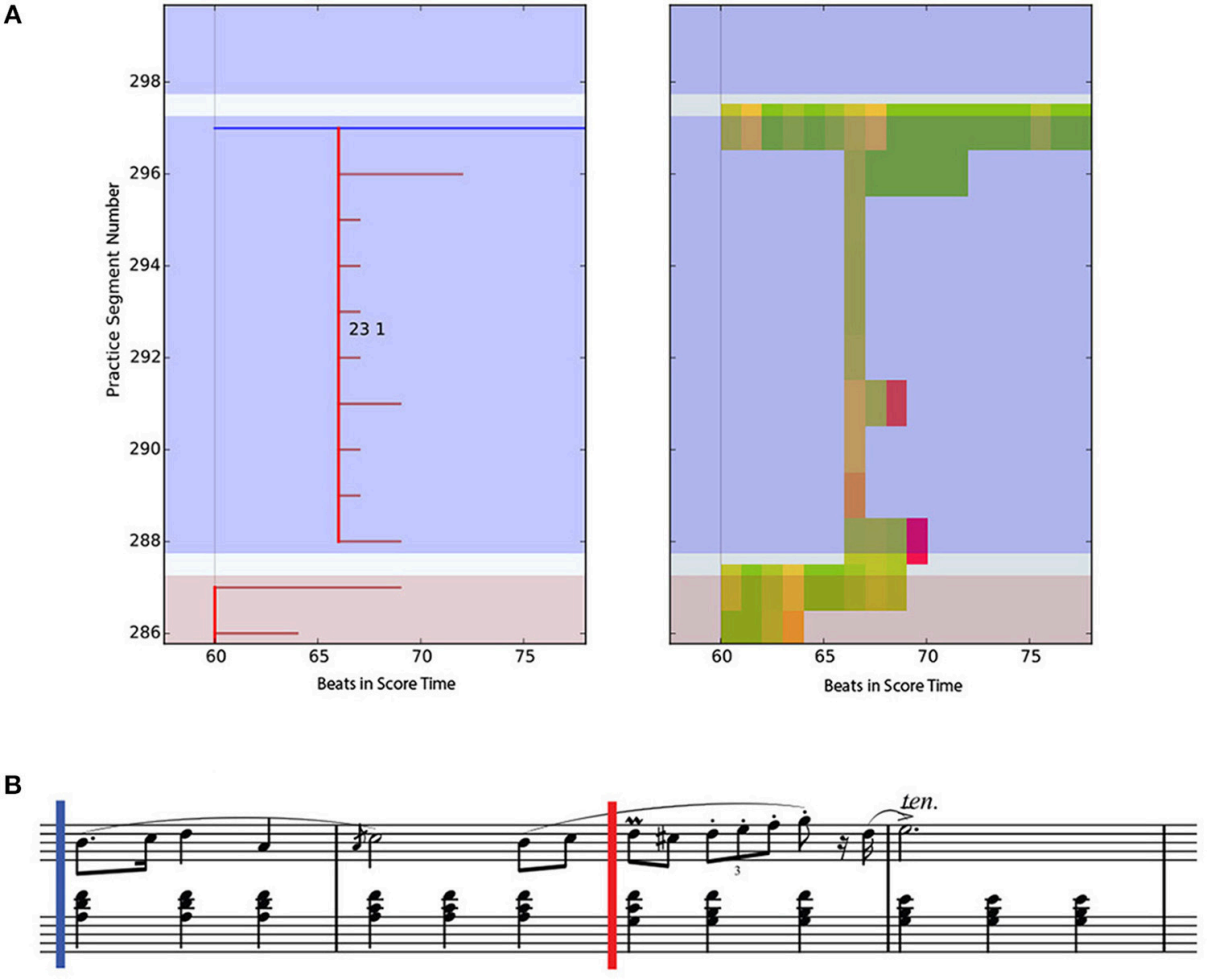
Practice Session Work Map (top left) and Practice Session Precision Map (top right) of a Drill-Smooth practice pattern of an intermediate participant (I-1) together with the score; corresponding music segment is shown in the score (lower part of figure). **(A)** Left - Practice Session Work Map with color annotations of drills (dark red) and smooth (blue). Right - Practice Session Preci- sion Map with color annotations of correct (green) and incorrect (red) percentage of notes in a beat. **(B)** Corresponding score. The point of interest starts at the red vertical line (m. 23 beat 1).

Whereas all practice segments begin from the same beat in a DS cluster, in a DS cluster, the last practice segment jumps back to before the start of the previous segment. A typical DS pattern is shown in Figure 12. Here, the musician was concerned with ornamentation of m. 23, which starts at the red vertical line in the score in Figure 12B. Note that the last practice segment in this cluster starts from m. 21, consolidating the work done in these parts of the piece. A PSPM is displayed on the right-hand side of the figure. Observe that the number of incorrect (red) notes diminishes over the course of the cluster.

The procedure for calculating DS clusters in the PSWM is similar to that for the DS clusters, except that the last segment should start at least 4 beats before time *t*_*i*_, including the beat that starts the cluster. The threshold of 4 beats was chosen because, for this piece, the smallest motif was one bar long, and we wanted to cover at least the motif length.

**Figure 13:**
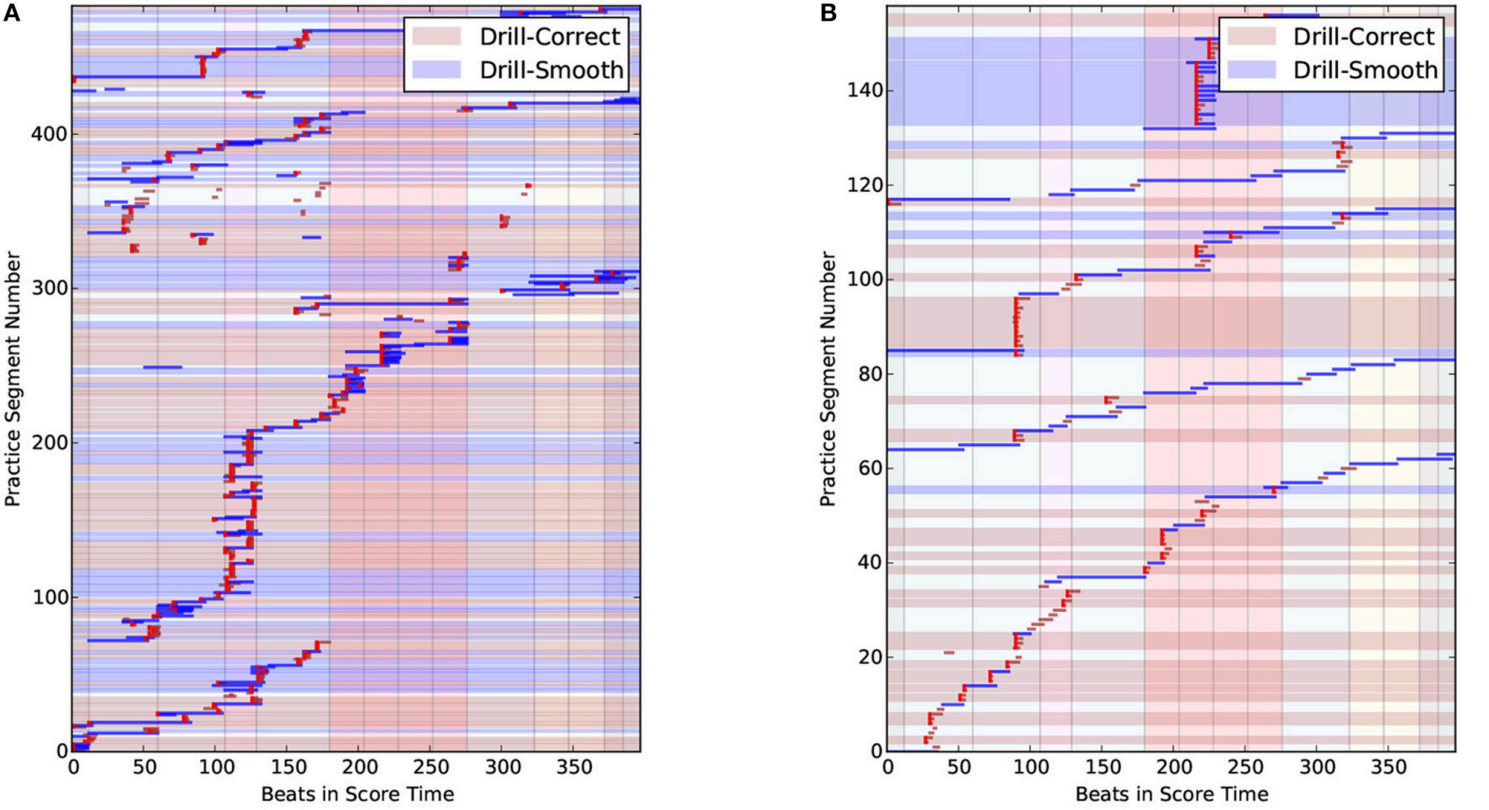
Practice Session Work Maps for E-2 (Left) and I-2 (Right) showing Drill-Correct clusters (light brown background) and Drill-Smooth clusters (light blue background). **(A)** PSWM with drill clusters for E-2. **(B)** PSWM with drill clusters for I-2.

Figure 13 shows two types of drill clusters in the PSWM. DS clusters are marked with a light dark red background, and DS clusters are indicated with a light blue background. The background of other practice patterns that did not fall into these two categories is left blank.

#### 5.1.3. Distribution of drill-correct and drill-smooth clusters

**Figure 14:**
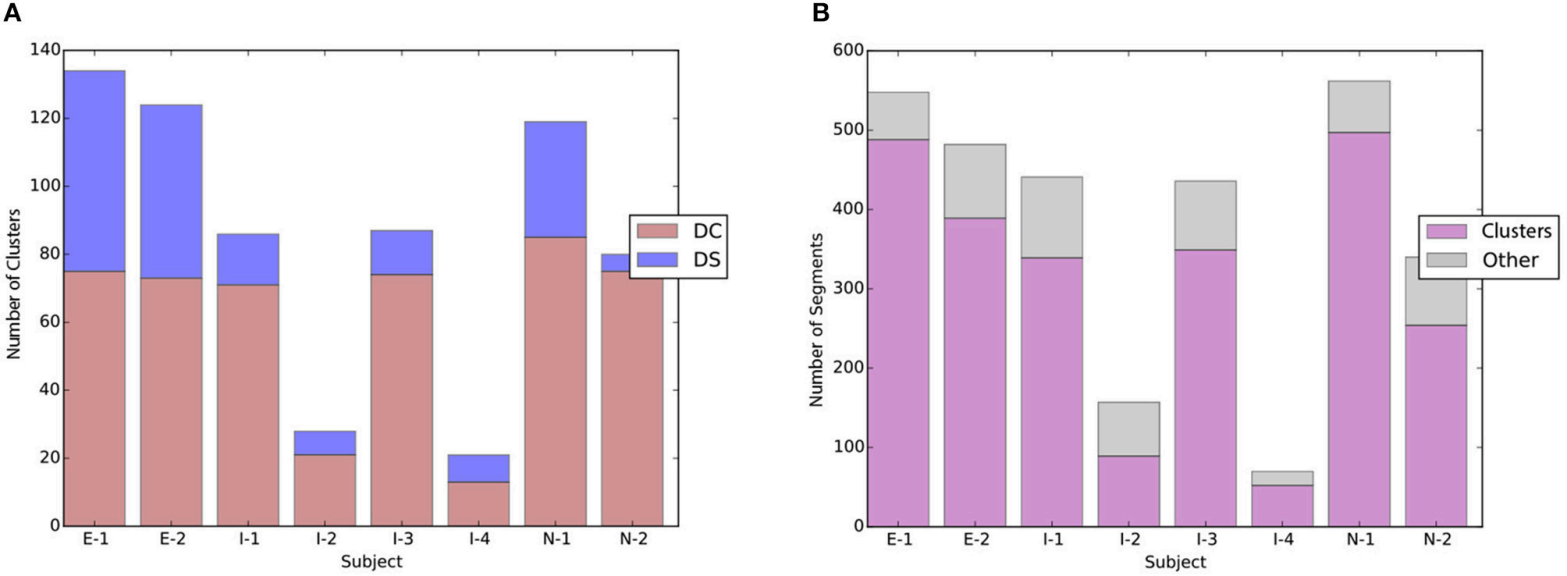
**(A)** Distribution of Drill-Correct vs. Drill-Smooth clusters in the experimental data; **(B)** distribution of DC/DS clusters vs. other practice patterns. **(A)** Ratio of Drill-Correct vs. Drill- Smooth clusters. **(B)** Ratio of DC/DS vs. other patterns.

Figure 14A shows the ratio of DS clusters vs. DS clusters for each participant. The height of the bar represents the total number of drill clusters for the participants. The blue part of the bar shows the number of DS clusters and the red part the number of DS clusters. It can be observed that all practice sessions contain both DC and DS clusters. DS clusters, both numerically and proportion-wise.

Based on our empirical data, the practice sessions consist mainly of drill clusters, see Figure 14B. Segments that do not belong to a drill cluster may belong to other practice pattern types such as *Review and Explore* as will be discussed in the next subsections.

### 5.2. Review and explore

As can be seen in Figure 14B, not all practice segments belong to a drill cluster. The second type of practice behavior that we observed in the study is the *Review and Explore* (RE) pattern. In RE practice patterns, the player consolidates previous work and looks for new ‘hazardous' places that might require their attention. While these segments can occur outside of drill clusters, the last practice segment of the latter can sometimes also be considered as a Review and Explore segment.

**Figure 15:**
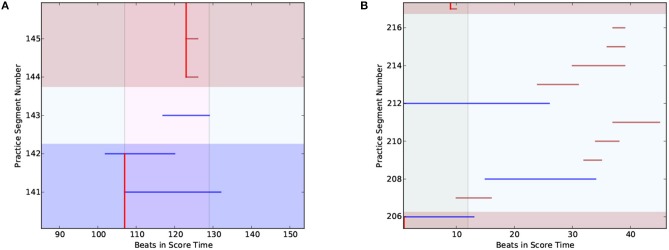
Examples of the review and explore practice pattern. **(A)** RE pattern (segments 142-143) from player I-2. **(B)** RE pattern (segments 206-216) from player N-2.

Figure 15 shows two types of RE patterns. On the left of the figure, segments 142 and 143 are Review and Explore patterns. Here, practice segment 142 is also the last segment of a DS cluster, but it also falls under the *Review* pattern category as it reviews previously practized work. The segment immediately following this (segment 143) belongs to the *Explore* pattern category as it looks ahead to new material that has not been practized before. The pianist in question, who is an expert pianist, stopped the Explore segment at the section boundary (beat 129), then jumped back to start a new drill cluster after noting the difficulty of the segment.

On the right of Figure 15 we have another Review and Explore pattern. This pattern was observed in the playing behavior of multiple intermediate and novice players. The figure shows a number of Explore patterns, each scouting for new material. In contrast to the behavior of expert pianists, the novices and beginners did not form a drill cluster after scouting, but continued to explore new material.

### 5.3. Memorization strategy

**Figure 16:**
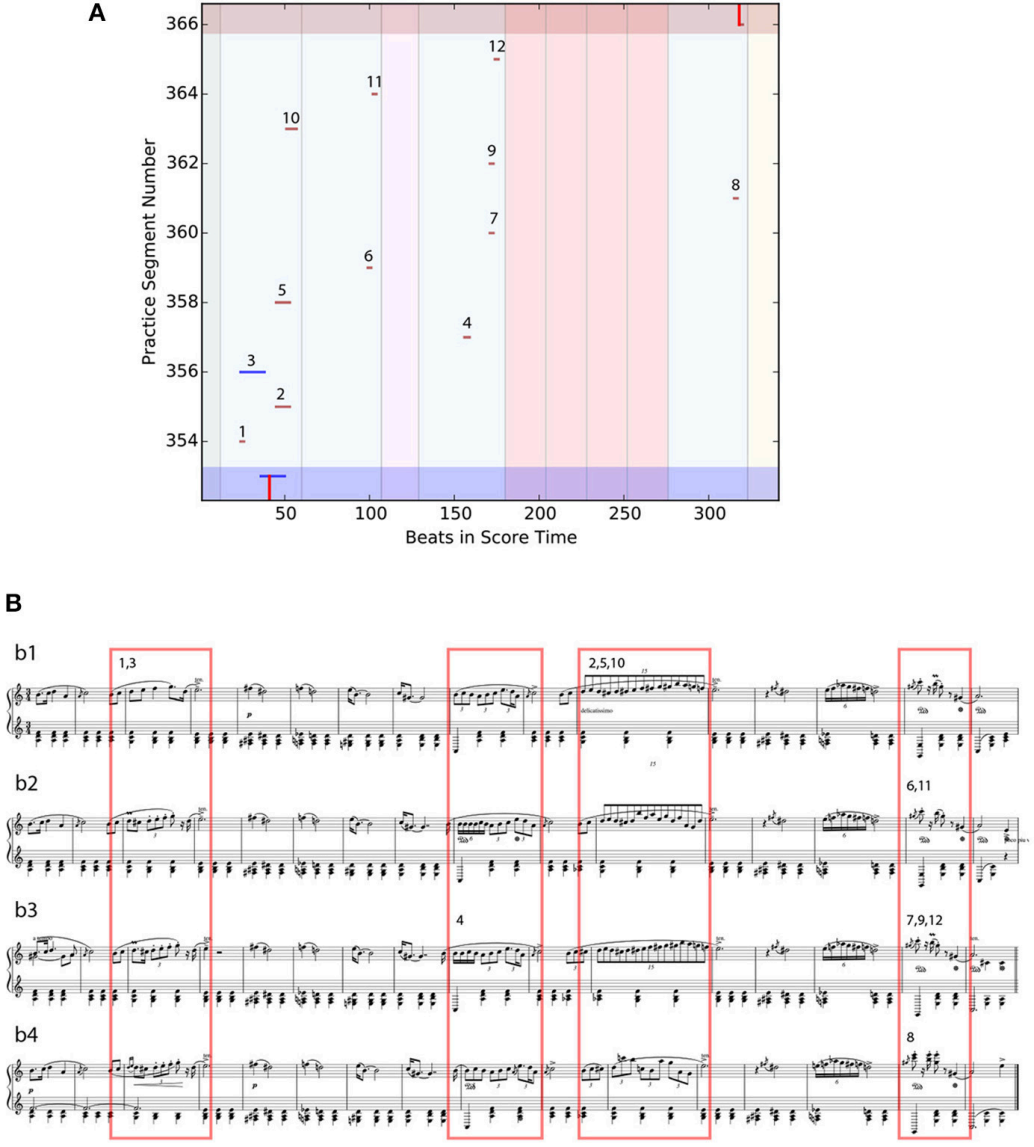
Representation of the Memorization practice pattern. Each number above a practice segment in the PSWM (Top) corresponds to the number inside of the rectangle in the score (Bottom). PSWM excerpt illustrating E-2's memorization strategy. **(A)** PSWM excerpt illustrating E-2's memorization strategy. **(B)** Corresponding score, showing the four similar subsections, *b1, b2, b3, b4* (see Figure 6). Red boxes show parts repeated with variations. Numbers link score fragments to practice segments.

Another practice pattern observed in the study is the Memorization Strategy (see Figure 16). This pattern can be seen in the PSWM of expert musician E-2, and only occurred once in the whole experiment. We interviewed the participant and asked about this practice pattern after the study, and learned that this was a memorization technique for locating small differences in similar sections. The differences in question are highlighted with red rectangles in Figure 16B. The PSWM clearly shows that the pianist played a number of seemingly unrelated short fragments. Upon further inspection we can see that the participant is playing fragments from similar sections. Each number above a practice segment in the PSWM (Figure 16A) corresponds to the number inside of the rectangle in the score (Figure 16B).

### 5.4. Expressivity of novices vs. advanced players

**Figure 17:**
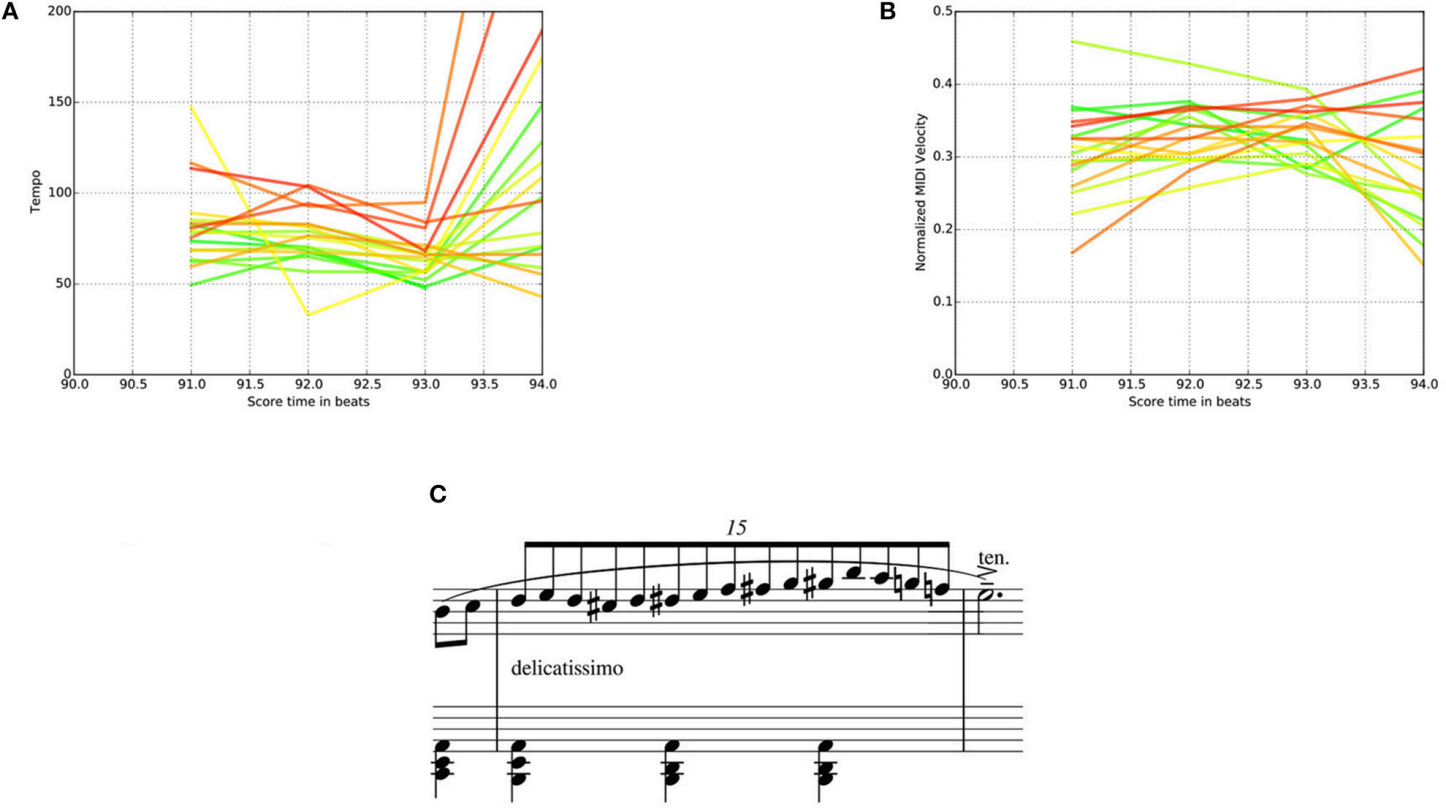
Tempo-Loudness Evolution Graphs for expert player E-1, for both loudness and tempo (Top) of a short music fragment (beats 90–94) (Bottom). Time progression is shown through the color of the plots, which range from green (beginning of practice session) to red (end of practice session). **(A)** Tempo-Loudness Evolution Graph for tempo. **(B)** Tempo-Loudness Evolution Graph for loudness. **(C)** Corresponding score.

Tempo-Loudness Evolution Graphs (TLEGs) can be used to track the progress of expressivity throughout a practice session. A visual example of how expressivity in a short music fragment, in terms of loudness and tempo, evolves throughout a practice session is displayed in Figure 17.

For the fast passage in m. 31 of the piece used in the experiment (see Figure 17C), the TLEG shows a clear increase in tempo over the course of the practice session. Indicating that E-1 practized this bar slowly at first (60 bpm) and gradually increased the tempo to around 100 bpm.

Advanced pianists typically have a good idea of the final performance, from the start of their practice (Chaffin et al., 2003). In this section, we investigate the similarity in expressivity between the practice average for each participant and the average of the MazurkaBL dataset.

**Figure 18:**
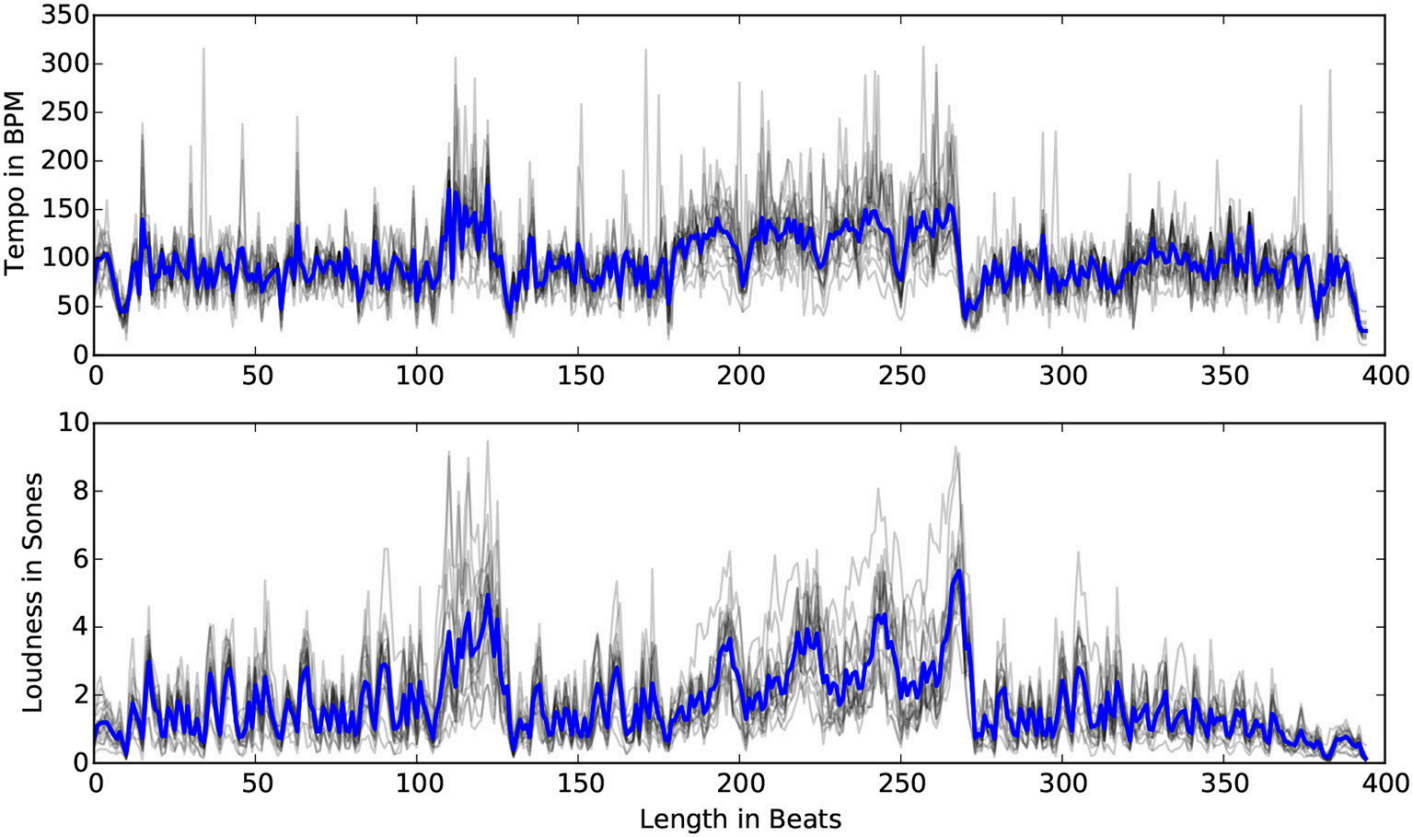
The average tempo and loudness (blue) for 46 performances in the Mazurka Dataset. Gray plots represent the data per pianist.

We used the dataset of beat and loudness annotations produced by Kosta et al. (2017).^9^ Recordings with automatic annotations above 300 bpm were removed, leaving a total of 46 recordings. Figure 18 shows the average loudness and tempo for the 46 recordings.

**Figure 19:**
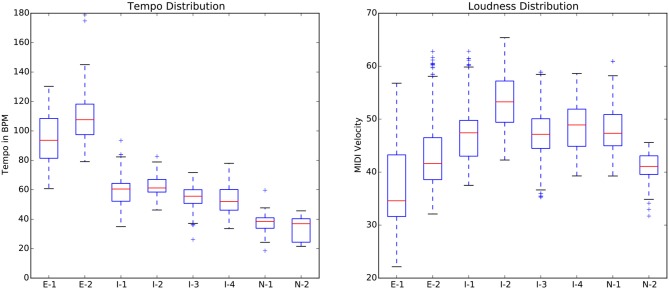
Box plots of distributions for tempo **(Left)** and loudness **(Right)** of the study. Red line shows the median; the top and bottom of the box show 75^th^ and 25^th^ percentile; whiskers indicate the interquartile range.

Returning to the empirical data from our experiment, the tempo and loudness of the practice sessions were calculated by taking average tempo and average loudness for each beat across practice segments for each individual. The box plots in Figure 19 show the spread of tempo and loudness parameters for each participant. The expert players' practice sessions showed higher variance (longer boxes) in both loudness and tempo as compared to novices. This indicates that they vary these parameters more throughout the practice session. They also play faster than novices, and while they have a larger loudness range, they play softer on average.

**Figure 20:**
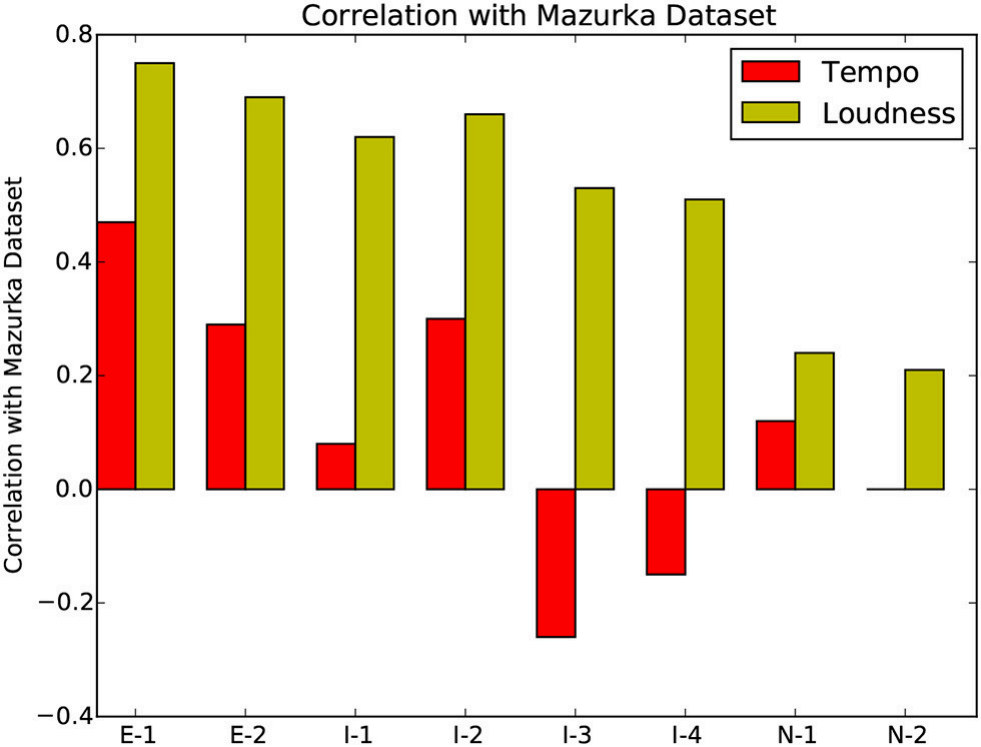
Pearson product-moment correlation coefficient between the expressive features of each participant with the average Mazurka dataset.

Table 1 gives the Pearson product-moment correlation coefficient between each participant's data and the corresponding tempo or loudness average for the aforementioned Mazurka dataset. A positive correlation indicates that the overall tempo/loudness shapes are similar to the Mazurka dataset average, with higher correlation for experts, as shown in Figure 20.

**Table 1:** Summary statistics for expressive parameters.

**Tempo (BPM)**	**Avg**	**Std**	**Min**	**Max**	**Corr**	***p*-value**
E1	95.15	14.56	60.76	130.28	0.469	< 1.0 × 10^−5^
E2	108.26	15.36	79.08	178.82	0.292	< 1.0 × 10^−5^
I1	58.84	8.45	34.95	93.45	0.081	0.112
I2	63.09	6.03	46.25	82.69	0.301	< 1.0 × 10^−5^
I3	54.92	7.22	26.13	71.70	–0.258	< 1.0 × 10^−5^
I4	52.31	8.92	33.61	77.98	–0.154	0.003
N1	37.77	5.64	18.52	59.67	0.121	0.017
N2	32.79	8.01	21.54	45.67	0.002	0.970
**Loudness (MIDI)**	**Avg**	**Std**	**Min**	**Max**	**Corr**	***p*****-value**
E1	36.4	7.23	22.13	56.79	0.748	< 1.0 × 10^−5^
E2	43.13	6.72	32.10	62.77	0.693	< 1.0 × 10^−5^
I1	47.29	5.48	37.5	62.81	0.619	< 1.0 × 10^−5^
I2	53.09	5.5	42.28	65.39	0.661	< 1.0 × 10^−5^
I3	46.91	5.07	35.25	58.87	0.525	< 1.0 × 10^−5^
I4	48.18	5.09	39.26	58.61	0.509	< 1.0 × 10^−5^
N1	48.18	5.09	39.26	60.92	0.241	< 1.0 × 10^−5^
N2	40.92	2.88	31.71	45.6	0.214	2.5 × 10^−5^

The *p*-values in the right-most column in Table 1 show the loudness correlations of all participants, and the tempo correlations of almost all participants, to be significant. The experts' playing are consistently and significantly correlated with the Mazurka average for both tempo and loudness. Only intermediate player I1 and novice player N2's tempo values are not significantly correlated with the average of the recorded performances. This confirms the idea that expert and intermediate players focus more on performance expressivity during practice, in order to better prepare for performances, while novices often do not have adequate control over quiet vs. loud and fast vs. slow playing.

## 6. Conclusions

In this research, we introduce a novel framework for musical practice visualizations, that integrates four novel visualization techniques: Practice Session Work Maps, Practice Precision Maps, Practice Segment Arcs, and Tempo-Loudness Evolution Graphs. These are then used in an empirical experiment to identify common practice behaviors. The consolidation of different types of visualizations makes MPB an ideal tool for both music teachers and students to critically analyse their practice behavior.

The first visualization technique implemented in MPB, the Practice Session Work Map, augments existing work Miklaszewski (1989); Chaffin and Imreh (2001) by integrating color-codes to distinguish between different types of practice patterns. They also allow us to visualize how practice segments develop in relation to the formal structure of the piece.

Practice Session Precision Maps, the second type of visualization, gives us an overview of the evolution of player's accuracy throughout the practice session. In contrast to existing software such as Wolfie and Superscore, MPB is the first visualization to provide playing accuracy information over the whole practice session in one graph.

Third, Practice Segment Arcs allow the user to easily visualize the practice flow and deduce difficult parts in the score. These diagrams allow the player to review difficult places or may assist teachers in suggesting techniques specifically targeted at these problematic fragments.

Finally, Tempo-Loudness Evolution Graphs offer a unique way to track the progression of expressivity, an oft-ignored characteristic in educational piano tools, throughout a practice session. After conducting several semi-structured interviews with four independent experts, one of whom was a participant in the study, three out of four found TLEGs useful. Two of the interviewees suggested that TLEGs could be used for comparing practice behaviors between musicians. One of the participants suggested that it would be good to learn from expert musicians' practice strategies, She also suggested that these graphs give students the ability to compare their own expressive gestures in practice session with those in commercial recordings.

Two interviewees found TLEGs to be complex and better suited for review under the supervision of a teacher. In such instances, the teacher could also use the TLEGs to monitor students' progress. One interviewee considered TLEGs to be useful for beginners since more advanced musicians should or would develop their own style of playing.

The combined feedback suggest that the Music Practice Browser could enhance online music education social networks such as PRAISE^10^ by dissecting and providing the details of students' practice behaviors.

In a future study, more general conclusions about practice patterns can be drawn using a larger number of participants. More variables, such as rubato, loudness and note-correctness could also be used to identify practice patterns. We will also explore how MPB can contribute to the learning experience if the visualizations are available immediately after a practice session. In this case, students would have immediate access to feedback on their practice patterns and behaviors, which may shape the direction of their future practice and stimulate them to practice more smartly and expressively.

The quality of the visualizations in MPB currently depend highly on the quality of the beat annotations; a small error in a beat annotation will reflect in incorrect precision in PSPMs. Factors such as hand-asynchrony, asynchrony of chords, and expressive variations all contribute to the accuracy. Here, all of the visualizations have been produced with the use of manual annotations in order to obtain the best possible accuracy. This manual process is time consuming as MPB needs both practice segment and beat annotations. Automatic alignment (Dannenberg, 1984; Raphael, 2001; Cont, 2008; Maezawa et al., 2011) could be used to expedite this process in future implementations. Such automatic methods have struggled with practice scenarios that may contain many errors, pauses, and repetitions. Recent research by Nakamura et al. (2016) and Sagayama et al. (2014) has aimed to tackle the problem of alignment of performances with incorrect notes. An alignment system trained especially on practice data containing tempo changes, restarts, and incorrect notes could be integrated into the MPB framework in the future.

In summary, an empirical study was conducted to evaluate the above-mentioned visualization tools for observing practice patterns in novice, intermediate, and expert pianists. We were able to identify and observe different types of practice patterns, including drill clusters, Explore and Review behaviors, a Memorization Strategy, and the Evolution of Expressivity during practice sessions.

In conclusion, this paper has proposed and presented a music practice pattern discovery tool. We have demonstrated that the tool is capable of revealing important practice patterns that may be used in the future as a diagnostic tool for piano learning. It is our intention the novel visualizations we have proposed may offer new avenues to understanding, and to prescribing treatments for, specific problems in piano learning. Further studies like that in Simones et al. (2017) can then investigate and validate the efficacy of the tool in piano learning environments.

## Ethics statement

Ethics committee-Queen Mary Ethics of Research Committee. Consent procedure-The participants were asked to sign a consent form where they agreed that the research study was explained to their satisfaction. At any point in time, the participant was free to leave the experiment. The participants agreed that personal information will be treated as strictly confidential and handled in accordance with the provisions of the Data Protection Act 1998. This study was carried out in accordance with the recommendations of Ms Hazel Covill, Queen Mary Ethics of Research Committee. The protocol was approved by the Queen Mary Ethics of Research Committee, ethics approval number: QMREC1585. All subjects gave written informed consent in accordance with the Declaration of Helsinki.

## Author contributions

JS implemented MPB, conducted the study and performed the initial data analysis. DH and EC contributed to the study design; data analysis and interpretation; and writing and editing of the paper.

### Conflict of interest statement

The authors declare that the research was conducted in the absence of any commercial or financial relationships that could be construed as a potential conflict of interest.

## Footnotes

^1^tonara.com

^2^timewarptech.com

^3^bandpad.co/en

^4^www.iiia.csic.es/praise

^5^www.apple.com/uk/logic-pro

^6^www.ableton.com

^7^lmms.io

^8^www.synthesiagame.com

^9^github.com/katkost/MazurkaBL

^10^www.iiia.csic.es/praise

## Abbreviations

m.: measure (bar)
mm.: measures (bars)
MPB: Music Practice Browser
PSM: Practice Session Map
PSPM: Practice Session Precision Map
PSWM: Practice Session Work Map
TLEG: Tempo-loudness evolution graph
RE: Review and Explore
DC, Drill-Correct: DS: Drill-Smooth.
